# Histopathological Differences Between De Novo and Nevus-Associated Melanoma

**DOI:** 10.3390/jcm15166399

**Published:** 2026-08-19

**Authors:** Alina Florentina Vasilovici, Mihai Gabriel Zaiț, Loredana Ungureanu, Alecsandra Iulia Grad, Răzvan M. Cherecheș, Carmen Georgiu, Simona Corina Șenilă

**Affiliations:** 1 Department of Dermatology, “Iuliu Hațieganu” University of Medicine and Pharmacy, 400012 Cluj-Napoca, Romania; 2 Department of Dermatology, Cluj-Napoca County Emergency Clinical Hospital, 400006 Cluj-Napoca, Romania; 3 Faculty of General Medicine, “Iuliu Hațieganu” University of Medicine and Pharmacy, 400012 Cluj-Napoca, Romania; mihaigabriel.zait@gmail.com; 4 Discipline of Pharmacology, Toxicology and Clinical Pharmacology, Faculty of Medicine, “Iuliu Hațieganu” University of Medicine and Pharmacy, 400012 Cluj-Napoca, Romania; 5 Department of Dermatology, “Dr. Constantin Opriș” Emergency County Hospital, 430031 Baia Mare, Romania; 6 HIVE Health Innovation Center, Babeș-Bolyai University, 400095 Cluj-Napoca, Romania; 7 Discipline of Histology, Department of Morphological Sciences, “Iuliu Haţieganu” University of Medicine and Pharmacy, 400012 Cluj-Napoca, Romania; 8 Department of Pathology, Emergency County Hospital, 400006 Cluj-Napoca, Romania

**Keywords:** cutaneous melanoma, nevus-associated melanoma, de novo melanoma, Breslow thickness, melanomagenesis, histopathology, clinicopathological features

## Abstract

**Background/Objectives:** Cutaneous melanoma arises de novo in the majority of cases; 20–30% develop from pre-existing nevi. Differentiating nevus-associated melanoma (NAM) from de novo melanoma (DNM) is important for understanding melanomagenesis, risk stratification, and prognostic assessment. The objective of this study was to compare the demographic, clinical, and histopathological characteristics of NAM and DNM in a large single-institution retrospective cohort. **Methods:** A retrospective, longitudinal, observational study was conducted at the Department of Pathological Anatomy, Cluj-Napoca County Emergency Clinical Hospital (2015–2025). Of the 729 initially identified cases, 580 patients with histopathologically confirmed cutaneous melanoma and complete clinical records were included. Analyses comprised Mann–Whitney U and chi-square tests and multivariate logistic regression (*p* < 0.05). **Results:** DNM constituted 78.6% (n = 456) and NAM 21.4% (n = 124) of cases. DNM patients were significantly older (median 66 vs. 56 years; *p* < 0.001). NAM was predominantly located on the trunk (63.7% vs. 45.2%; *p* = 0.0006), whereas DNM showed greater involvement of the head/neck and upper extremities. Superficial spreading melanoma was more frequent in NAM (79.8% vs. 62.5%; *p* = 0.004); nodular melanoma was more common in DNM (22.6% vs. 10.5%), and lentigo maligna was exclusive to DNM. DNM demonstrated greater Breslow thickness (median 2.0 vs. 1.25 mm; *p* = 0.021), more advanced Clark level (*p* = 0.040), and higher mitotic rate (median 3 vs. 2/mm^2^; *p* = 0.049). On multivariate logistic regression, older age (OR = 1.03; 95% CI: 1.02–1.05; *p* < 0.001) and head/neck localisation (OR = 2.73; 95% CI: 1.39–5.39; *p* = 0.004) were independent predictors of DNM; histopathological aggressiveness markers did not retain independent significance after adjustment. **Conclusions:** Although DNM presents with more aggressive histopathological features, these characteristics are driven primarily by patient age and anatomical location rather than an inherent de novo aggressive phenotype, suggesting that observed differences largely reflect cumulative sun-damage pathways and patient demographics.

## 1. Introduction

Cutaneous melanoma is a malignancy of increasing global incidence and the most common cause of skin cancer mortality. According to GLOBOCAN 2022, melanoma was the 17th most common cancer worldwide, with an estimated 331,722 new cases and 58,667 deaths [1]. Most melanomas arise de novo, on previously normal-appearing skin, whereas approximately 20–30% develop in association with a pre-existing melanocytic nevus [2,3,4,5,6]. Distinguishing these two groups is of particular interest for understanding melanomagenesis and for its implications in risk stratification, targeted screening, and prognosis [2,7,8].

Recent population-based and large retrospective studies have clarified the clinicopathologic and molecular differences between nevus-associated melanomas (NAMs) and de novo melanomas (DNMs) [9]. NAMs tend to occur in younger individuals, are more frequently located on the trunk, and are associated with a higher total nevus count [3,4,5,10,11]. DNMs tend to display more aggressive features, often presenting with greater Breslow thickness and a more advanced stage at diagnosis than NAMs [4,6,7,8,12,13]. The presence of a pre-existing nevus, frequently inconspicuous clinically and dermoscopically, can complicate histopathological assessment, requiring careful examination for subtle architectural or cytological alterations indicative of malignant transformation [14,15,16,17,18,19]. A nevus-associated melanoma is defined by the concurrent presence of both a melanocytic nevus and a melanoma within the resected specimen, confirmed by histopathological analysis [11,12].

NAM and DNM differ with respect to their underlying oncogenic drivers, histological subtype distribution, patient demographics, and risk factors, and these differences carry direct implications for clinical management. Patients with NAM—generally younger and with a higher nevus count—may benefit from dermoscopic monitoring of their remaining nevi, while those with DNM—typically older and presenting at chronically sun-exposed sites—are better suited to photoprotection measures and age-adapted surveillance programmes [2,3,4].

Molecularly, NAM is characterised by a higher prevalence of BRAF V600E mutations, a defining feature of the proliferative, non-cumulative sun damage (non-CSD) pathway, whereas DNM shows a greater frequency of NF1 loss-of-function and TERT promoter mutations, reflecting UV-driven, CSD-associated mutagenesis; NRAS mutations are seen in both subtypes [9]. These molecular differences help explain the distinct epidemiological and histological patterns observed and form the biological basis for distinguishing NAM from DNM in clinical practice and research.

The two-phase growth model helps explain the differing subtype distribution between the two groups. Superficial spreading melanoma (SSM), which predominates in NAM, is marked by an extended radial growth phase with horizontal spread within the epidermis, allowing a longer interval during which clinical and dermoscopic detection is possible before vertical invasion begins. Nodular melanoma (NM), more common in DNM, generally transitions to vertical growth early on with little or no radial phase, leading to rapid infiltration of the dermis, greater tumour thickness, and a more aggressive clinical trajectory [20,21]. This growth-phase model may help account for why DNM tends to present with greater Breslow thickness and higher mitotic rates at the time of diagnosis [4,12].

Survival decreases significantly with increasing Breslow thickness, underscoring the prognostic importance of accurate differentiation between these subtypes [22]. The 2018 World Health Organization (WHO) Classification of Skin Tumours integrates clinical, epidemiological, pathological, and molecular features to define distinct pathways of melanoma pathogenesis, highlighting the complexity of distinguishing de novo lesions from those arising within pre-existing nevi [20]. This framework emphasises the need for meticulous histopathological examination, particularly where early-stage melanoma and benign nevi show overlapping features, especially in acral regions [21,23].

The primary aim of this study was to determine whether de novo and nevus-associated cutaneous melanomas exhibit distinct clinicopathological profiles in a large single-centre Romanian cohort and to identify independent demographic and histological predictors of de novo origin using multivariable logistic regression. Secondarily, we aimed to assess the prevalence of NAM and to examine its distribution across demographic, clinical, and histopathological characteristics compared with DNM.

## 2. Materials and Methods

This single-centre, retrospective, observational, cross-sectional study included patients diagnosed with cutaneous melanoma at the Department of Pathological Anatomy of the Cluj-Napoca County Emergency Clinical Hospital (SCJU Cluj-Napoca), a tertiary institution that serves as the regional referral centre for the North-West development region, between 2015 and 2025. A total of 729 patients with clinical suspicion and histopathological confirmation of melanoma were identified from the departmental database. Of these, 149 were excluded (patients under 18 years of age, incomplete medical records, recurrent or metastatic lesions, and non-cutaneous melanomas), leaving 580 complete records for analysis.

Inclusion criteria were a histopathologically confirmed diagnosis of cutaneous melanoma and complete, verified clinical data (age, sex, lesion location, Breslow thickness, Clark level, mitotic rate, ulceration, tumour regression, final histological diagnosis, de novo versus nevus-associated origin, and lymphatic, vascular, and perineural invasion, and resection-margin status).

Excised specimens were fixed in 10% neutral buffered formalin, measured, and entirely submitted for histological examination; specimens were serially sectioned at 2–3 mm intervals (bread-loaf technique), and all sections were paraffin-embedded, with multiple tissue levels examined per block to maximise detection of residual nevus elements. Routine histological assessment was performed with haematoxylin and eosin (H&E) staining. Immunohistochemistry was not applied systematically. It was performed at the reporting pathologist’s discretion whenever the H&E appearances were equivocal, when the distinction between melanoma and a benign melanocytic proliferation was in question, when the presence or depth of dermal invasion required confirmation, or when tumour cell lineage was uncertain in a poorly differentiated lesion. Complementary immunohistochemical studies were documented in 177 of the 580 reports (30.5%)—in 132 of 456 de novo cases (28.9%) and 45 of 124 nevus-associated cases (36.3%). The markers used, in descending order of frequency, were HMB-45 (n = 143), Ki-67 (n = 86), Melan-A/MART-1 (n = 74), SOX10 (n = 41), S100 protein (n = 40), PRAME (n = 38), p16 (n = 31), and cytokeratins (n = 11). Each case was reported by a single board-certified pathologist. Cases judged diagnostically equivocal by the reporting pathologist were reviewed by a second departmental pathologist before the report was issued, in accordance with routine departmental practice. There was no systematic independent double reading of the whole series, and no blinded re-review of archival slides was performed for the purposes of the present study; consequently, no formal measure of inter-observer agreement (such as Cohen’s κ) can be reported. Reporting pathologists had access to the clinical diagnosis stated on the request form but did not have routine access to clinical photographs or to dermoscopic images. No clinical photograph was referenced in any of the 580 reports, and a dermoscopic diagnosis was mentioned in the referral information in 118 cases (20.3%).

**Figure 1:**
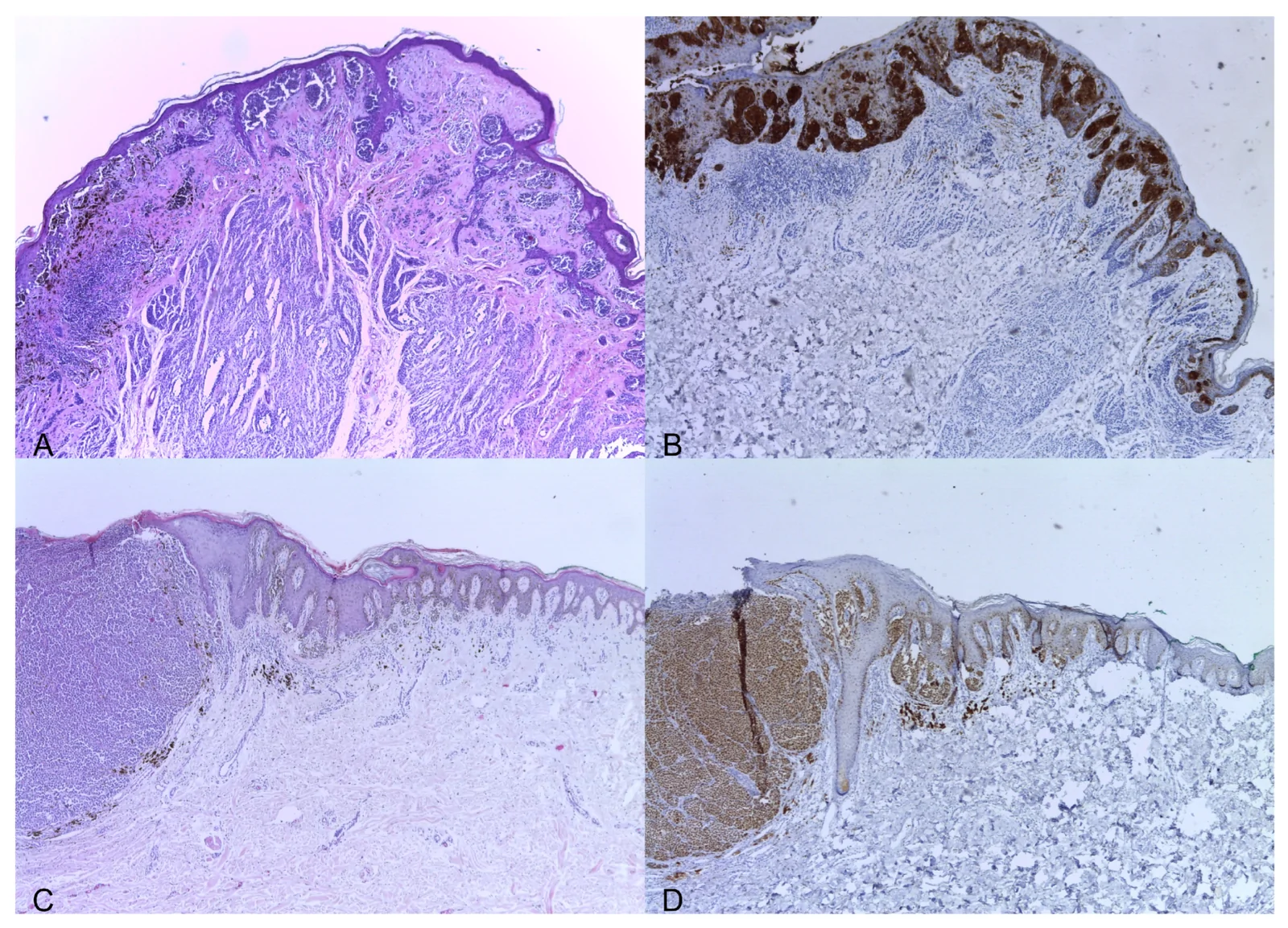
Histopathological comparison of nevus-associated and de novo melanoma: (**A**,**B**) Nevus-associated melanoma: (**A**) Superficial spreading melanoma in situ, showing pagetoid spread of atypical melanocytes throughout the epidermis; the underlying dermis contains a population of benign melanocytes showing maturation with descent, corresponding to a compound melanocytic nevus. (**B**) The same lesion stained for HMB-45: the intraepidermal and junctional malignant melanocytes are immunoreactive, whereas the benign neval melanocytes in the dermis are negative. (**C**,**D**) De novo melanoma: (**C**) Melanoma in the vertical growth phase (**left** of the field), with an adjacent junctional component showing superficial spreading growth (**right** of the field); no residual nevus component is identifiable. (**D**) PRAME immunohistochemistry of the lesion shown in (**C**): all malignant melanocytes show nuclear positivity. Haematoxylin and eosin staining (**A**,**C**); immunohistochemistry (**B**,**D**). All images were acquired with a ×4 objective.

Lesions were classified as nevus-associated melanoma (NAM) when a melanocytic nevus and a melanoma were histopathologically contiguous within the same specimen, and as de novo melanoma (DNM) otherwise (Figure 1).

Operationally, the criterion applied was the presence, at the periphery of or immediately beneath the malignant proliferation, of a population of cytologically bland melanocytes showing maturation with descent into the dermis and arranged in the architectural pattern of a common acquired, dysplastic, or congenital-pattern nevus, sharply demarcated from the adjacent malignant population. Nevi that were separate from the melanoma within the same specimen, without direct continuity, were not counted as nevus-associated. DNM is defined here by the absence of an identifiable precursor rather than by positive evidence that no precursor ever existed. The category “Other” histological subtype comprised epithelioid cell melanoma (4 cases), mixed epithelioid and spindle cell melanoma (3 cases), regressing melanoma (2 cases), desmoplastic melanoma (3 cases), spitzoid melanoma (1 case), and spindle cell melanoma (1 case).

The study was approved by the Ethics Committee of the Cluj-Napoca County Emergency Clinical Hospital (approval No. 44950/09.12.2025) and Iuliu Hațieganu University of Medicine and Pharmacy (approval No. AVZ51/05.02.2026), which authorised the retrospective use of anonymised archival pathology records for research; given the retrospective design based on de-identified existing records, individual written informed consent was covered by this approval. Data were handled in accordance with the General Data Protection Regulation (GDPR): patient names were never extracted, and the medical-record number was retained only transiently for database verification and deleted upon completion of that verification, after which only age and sex remained as extracted variables.

Statistical analysis was performed in Jamovi 2.7.28. Descriptive statistics are reported as the median for continuous variables and as frequencies (percentages) for categorical variables. Medians are accompanied by the interquartile range [IQR]. The distribution of continuous variables was assessed with the Kolmogorov–Smirnov test; as variables deviated from normality, between-group comparisons used the non-parametric Mann–Whitney U test. Categorical variables were compared with the χ^2^ test or Fisher’s exact test (for expected cell counts < 5, including the rare micro-staging variables). Independent predictors of de novo origin were identified by multivariable binary logistic regression, with de novo origin as the outcome and nevus-associated origin as the reference category. Candidate predictors were pre-specified on clinical and biological grounds before the model was fitted. The pre-specified set comprised the two demographic variables (age, entered as a continuous linear term in years, and sex), anatomical site (five categories, trunk as reference), histological subtype (superficial spreading melanoma as reference), and the two histopathological markers whose independence from age and site constituted the primary question of the study (Breslow thickness and mitotic rate, both continuous). No interaction terms were specified, and no variable was removed after fitting. Statistical significance was set at a two-sided *p*-value below 0.05.

Artificial Intelligence Assistance: During the preparation of this work, Claude (Opus 4.x; Anthropic PBC, San Francisco, CA, USA), accessed via the Reactor AI platform (May 2026), was employed to assist with the synthesis of the published literature and with language editing of selected manuscript passages. The tool was not involved in study design, data collection, statistical analysis, or the formulation of results or conclusions. All AI-generated content was independently reviewed, critically assessed, and revised by the authors, who accept full responsibility for the accuracy, integrity, and completeness of the published work. No AI system is listed as an author.

## 3. Results

### 3.1. Demographic Characteristics and Patient Distribution

The cohort comprised 580 patients with surgically excised cutaneous melanoma. By tumour origin, 456 (78.6%) were classified as DNM and 124 (21.4%) as NAM, a ratio of approximately 3.7:1. Age at diagnosis differed significantly between groups (*p* < 0.001, Mann–Whitney U): the median age was 66 [53–74] years in the DNM group (mean 63.9 ± 15.1) versus 56 [44–66] years in the NAM group (mean 55.8 ± 15.2). Sex distribution was balanced, with no significant difference between groups (*p* = 0.634, χ^2^). Demographic and anatomic location data are summarised in Table 1.

### 3.2. Anatomic Site Distribution

The anatomic distribution of lesions differed significantly between the two groups (*p* = 0.0006, χ^2^). NAMs were predominantly truncal (63.7%, n = 79) compared with 45.2% (n = 206) of DNMs. Head-and-neck localisation was more frequent in DNMs (24.3%, n = 111) than in NAMs (9.7%, n = 12), as was upper-limb localisation (11.8% vs. 6.5%). Lower-limb involvement was comparable between groups (17.3% vs. 18.5%).

### 3.3. Histological Subtypes and In Situ Lesions

Among histological subtypes, SSM predominated in both groups but was significantly more prevalent in NAMs (79.8%, n = 99) than in DNMs (62.5%, n = 285; *p* = 0.004). Nodular melanoma was more than twice as frequent in the DNM group (22.6%, n = 103) as in NAM (10.5%, n = 13). Lentigo maligna melanoma occurred exclusively in the DNM group (2.9%, n = 13). The proportion of in situ melanomas was comparable between groups (6.6%, n = 30 of DNM vs. 7.3%, n = 9 of NAM).

### 3.4. Histopathological Characteristics

Breslow thickness was significantly greater in DNM (median 2.00 [0.70–5.00] mm) than in NAM (1.25 [0.69–3.12] mm; *p* = 0.021, Mann–Whitney U), and the Clark level of invasion was more advanced in DNM (*p* = 0.040). The mitotic rate was higher in DNM (median 3 [1–9]/mm^2^) than in NAM (2 [1–6]/mm^2^; *p* = 0.049). A strong positive correlation was observed between Breslow thickness and mitotic rate across the cohort (Spearman ρ = 0.73). No significant differences were found for ulceration (42.8% vs. 34.7%; *p* = 0.128), regression (31.4% vs. 26.6%; *p* = 0.363), or perineural invasion (2.0% vs. 2.4%; *p* = 0.726). Histopathological characteristics are detailed in Table 2.

### 3.5. Micro-Staging and Resection Margins

Lymphatic invasion was identified in 7.9% of DNMs and 4.8% of NAMs, a difference that did not reach significance (*p* = 0.32). Vascular invasion occurred in fewer than 3% of cases overall, with no significant difference between groups or by anatomic site (*p* > 0.05). When analysed by histological subtype irrespective of DNM/NAM origin, perineural invasion was significantly associated with subtype (*p* < 0.001), being most frequent in acral lentiginous melanoma; lymphatic invasion showed a marginal association (*p* = 0.058), with higher rates in acral and nodular subtypes than in SSM.

Positive/incomplete resection margins (R1) were recorded in 5.7% of DNMs and 2.4% of NAMs (*p* = 0.16). The R1 rate varied significantly by anatomic location (*p* = 0.02; upper limbs 9.7% and head/neck 8.1% vs. trunk 2.1%) and by histological subtype (*p* = 0.002; “Other” subtypes 21.4% and nodular 9.5% vs. SSM 3.1%).

### 3.6. Solar Elastosis: Distribution by Anatomical Site and Histological Subtype

Solar elastosis was documented in 106 of 565 pathology reports in which the feature was explicitly recorded (18.8%). Its prevalence varied markedly by anatomical site (χ^2^ = 88.2; df = 3; *p* = 5.4 × 10^−19^): head and neck, 53/112 (47.3%); upper extremities, 5/39 (12.8%); trunk, 28/281 (10.0%); and lower extremities, 5/85 (5.9%). The head and neck region was associated with an approximately 8-fold higher likelihood of solar elastosis compared with the trunk (OR = 8.1; *p* = 3.3 × 10^−15^, Fisher’s exact test).

By histological subtype, lentigo maligna/LMM showed the highest prevalence of solar elastosis (12/13; 92%), followed by nodular melanoma (27/124; 22%), the “Other” category (17/83; 20%), melanoma in situ (3/17; 18%), and superficial spreading melanoma (47/305; 15%). Acral lentiginous melanoma showed no solar elastosis (0/23; 0%). When cases were stratified jointly by histological subtype and anatomical site, the anatomical gradient was maintained across all subtypes: among SSM, solar elastosis was recorded in 47% of head/neck lesions versus 9% of truncal lesions (OR = 9.2; *p* = 5.6 × 10^−8^). Within the head and neck region, solar elastosis was significantly more frequent in LMM (8/9; 89%) than in non-LMM subtypes combined (45/103; 44%; *p* = 0.013). These data are summarised in Table 3.

### 3.7. Multivariable Analysis

To identify independent predictors of de novo origin, a multivariable logistic regression model was fitted, adjusting for demographic, clinical, and histopathological variables (Table 4). Older age (OR 1.03 per year, 95% CI 1.02–1.05; *p* < 0.001) and tumour location on the head and neck (OR 2.73, 95% CI 1.39–5.39; *p* = 0.004) or upper limbs (OR 2.57, 95% CI 1.13–5.81; *p* = 0.024) were the strongest independent predictors of DNM. Histopathological variables that were significant in univariate analysis (Breslow thickness, mitotic rate) did not retain significance after adjustment (*p* > 0.05).

## 4. Discussion

The present analysis identifies a consistent set of clinicopathological differences between NAM and DNM in a large Romanian single-centre cohort. The DNM:NAM ratio of approximately 3.7:1 corroborates the widely held view that most cutaneous melanomas arise without a recognisable precursor lesion [2,3,4,6]. Our NAM proportion (21.4%) is consistent with recent cohorts, such as Dika et al. (24%) [4] and Olsen et al. (24.3%) [3]. By contrast, Kostaki et al. reported a substantially higher NAM proportion of 56.8% [24]; this difference is likely attributable to their younger study population, in whom nevus-associated lesions are proportionally more frequent.

A pronounced age disparity characterised the two groups. The substantially older median age of DNM patients (66 vs. 56 years) supports the hypothesis that de novo melanoma is in large part a consequence of cumulative UV-induced cutaneous damage accumulated over decades, while NAM more often manifests in younger individuals with a higher melanocytic nevus burden. This age-related pattern is consistent with published data linking de novo lesions to chronically sun-exposed anatomical sites and older patient age [5,7,12,13].

The predominance of NAMs on the trunk (63.7%) is compatible with the hypothesis that intermittent, high-intensity UV exposure typical of this region promotes the proliferation of melanocytic nevi, some of which may subsequently undergo malignant transformation. Conversely, the greater representation of DNMs on the head, neck, and upper limbs is consistent with the chronic sun-damage (CSD) pathway, in which cumulative UV exposure may drive malignant transformation of melanocytes without a clinically evident precursor [20]. Within the WHO framework, melanomagenesis is organised into distinct evolutionary pathways defined by specific genetic alterations, precursor-lesion types, and degrees of CSD; low-CSD melanomas (such as SSM, which may develop from a nevus or dysplastic nevus) are distinguished from high-CSD melanomas (such as lentigo maligna melanoma), which typically arise from melanoma in situ rather than a benign precursor [20]. Within this scheme, NAMs fall predominantly into the low-CSD category. The association of NAM with truncal location and younger age is in keeping with other cohorts [3,4,5,10,11,24].

The greater Breslow thickness and higher Clark level in the DNM group may reflect three complementary, non-exclusive mechanisms, which we frame as hypotheses rather than demonstrated causes. First, the enrichment of nodular melanoma in DNM—a subtype that enters vertical growth early with minimal or absent radial phase—directly contributes to greater thickness at diagnosis [20,21]. Second, the absence of a pre-existing “sentinel” nevus as a clinical reference point may delay patient vigilance and clinical attention relative to a changing pre-existing mole, potentially lengthening the preclinical interval [16,25]. Third, the older age profile of DNM patients is associated with diminished immune surveillance capacity, which may also contribute to a longer asymptomatic period before diagnosis [26]. The strong correlation between Breslow thickness and mitotic rate supports the internal consistency of the data, in that thicker tumours were also more proliferative.

In contrast to studies linking ulceration to specific aggressive subtypes, our data—showing no significant between-group difference (42.8% vs. 34.7%; *p* = 0.128)—are compatible with ulceration being largely a function of tumour thickness rather than an intrinsic feature of de novo origin. This finding aligns with Dika et al. (2025) [4], who similarly reported no significant difference in ulceration between DNM and NAM after adjustment for thickness. Regarding mitotic rate, the higher median rate in DNM (3 vs. 2/mm^2^; *p* = 0.049) is consistent with Cymerman et al. (2016) [13], who reported significantly higher mitotic rates in DNM in a large US cohort. The loss of independent predictive value of both parameters in our multivariate model indicates that they are biological correlates of age and anatomical location rather than independent markers of de novo origin.

The distribution of histological subtypes further supports this clinical dichotomy. The predominance of SSM in NAM (79.8%) is consistent with a lesion characterised by a prolonged radial growth phase that clinically overlaps with a pre-existing nevus. The roughly 2.75-fold higher frequency of nodular melanoma in DNM is consistent with the more aggressive behaviour of this subtype, which characteristically arises de novo through early vertical growth and may escape early clinical detection. The exclusive occurrence of lentigo maligna melanoma in the DNM group mirrors the CSD pathway, as this subtype classically arises on severely sun-damaged skin in older patients [20].

Interestingly, the proportion of in situ melanomas was comparable between groups (6.6% vs. 7.3%). This finding should not be interpreted as evidence that nevus-associated melanomas are not detected earlier through surveillance; the variables required to assess screening impact—surveillance modality, prior dermatologic follow-up, and interval from symptom onset to excision—were not available in this retrospective dataset. Surveillance modalities including total-body photography and digital dermoscopy follow-up have been associated with earlier-stage diagnoses in high-risk patients [24], and their potential contribution to stage distribution in NAM cannot be excluded from this analysis. The comparable in situ rates likely reflect mechanistically distinct pathways converging at a similar histological snapshot: in NAM, early pagetoid spread is sustained partly by *BRAF* V600E-driven intraepidermal proliferation, whereas in DNM (e.g., lentigo maligna in situ) it reflects *TERT* promoter-driven CSD mutagenesis without immediate dermal invasion [9]. Both pathways may sustain intraepidermal proliferation before the vertical-growth transition, resulting in similar in situ rates despite divergent oncogenic origins. This observation is hypothesis-generating and would require prospective, stage-stratified data for confirmation.

Although lymphatic invasion did not differ significantly between DNM and NAM, its numerically higher frequency in DNM may be a signal of metastatic potential and warrants confirmation in larger series. Analysis by histological subtype suggested higher rates of lymphatic dissemination in non-SSM subtypes (acral and nodular); however, given the small acral subgroup (n = 16), these estimates are imprecise and should be regarded as preliminary. Similarly, the association of perineural invasion with histological subtype suggests that neurotropism may be intrinsic to specific cellular lineages (particularly acral lentiginous melanoma) rather than a function of anatomic site, but this remains hypothesis-generating. These subtype findings should be interpreted with considerable caution: the acral subgroup comprised only 16 cases, rendering subtype-stratified estimates of lymphatic invasion and perineural invasion highly imprecise and statistically underpowered. The reported acral rate (18.8%, i.e., 3 of 16 cases) is based on very few events, and no definitive conclusions regarding differential invasion risk by histological subtype can be drawn from these data; they should be regarded as preliminary and hypothesis-generating.

The slightly higher rate of positive margins (R1) in DNM, although not statistically significant, may partly reflect the greater difficulty of clinically demarcating lesions that lack a recognisable associated nevus—a nevus component is clinically identifiable in approximately 48% of NAMs [25]. More specifically, lentigo maligna melanoma—exclusive to the DNM group and predominantly located at head and neck sites—is characterised by subclinical lateral extension and notoriously difficult margin demarcation; its exclusive occurrence in DNM is likely an important driver of the higher R1 rate in this group. The significant association of positive margins with head-and-neck and upper-limb locations more likely reflects these combined site-specific surgical complexities—limited tissue reserve and LMM morphology—than intrinsic tumour biological aggressiveness.

Solar elastosis—defined histologically as dermal accumulation of degraded elastic fibres resulting from chronic UV radiation—has historically been regarded as a hallmark of lentigo maligna/LMM and as a tissue-level proxy for the chronic sun-damage (CSD) pathway [20]. Our data confirm the strong and expected association with LMM (12/13; 92%) but demonstrate that solar elastosis is not confined to this subtype. It was detected in 22% of nodular melanomas, 15% of SSM cases, and 18% of in situ melanomas in our cohort, indicating that the pathological substrate of cumulative UV exposure extends across histological categories. Crucially, the dominant determinant of solar elastosis prevalence was anatomical site rather than histological subtype: head and neck tumours showed approximately 8-fold higher rates than truncal tumours (47.3% vs. 10.0%; OR = 8.1; *p* = 3.3 × 10^−15^). This gradient was maintained within SSM (head/neck 47% vs. trunk 9%; OR = 9.2; *p* = 5.6 × 10^−8^), providing compelling evidence that solar elastosis in non-LMM subtypes reflects the degree of cumulative, site-specific UV damage rather than an intrinsic property of any single histological category. The complete absence of solar elastosis in acral lentiginous melanoma (0/23; 0%)—a subtype arising on glabrous skin with minimal UV exposure—provides internal validation of this anatomical logic. Taken together, these findings reinforce the CSD/non-CSD framework: melanomas arising at chronically photo-exposed sites, regardless of histological subtype, share the tissue-level signature of cumulative solar damage. The occurrence of solar elastosis in a substantial proportion of SSM and nodular melanomas at head and neck sites supports the view that these tumours may engage CSD-associated pathogenetic mechanisms (enrichment of *NF1* loss-of-function and *TERT* promoter mutations) [9,20], even when classified as a “non-CSD” subtype on the basis of growth pattern alone. This observation is consistent with the epidemiological data from our cohort, in which the head/neck site and advanced patient age were the two independent predictors of de novo origin—both surrogates for cumulative UV exposure.

The multivariable model provides the most important refinement of the DNM profile. The loss of independent predictive value of the classical aggressiveness markers (Breslow thickness, mitotic rate) after adjustment for age and location is a key finding. This is consistent with the hypothesis that the aggressive features attributed to de novo melanoma are not independent causal factors but rather biological correlates of advanced patient age and of tumours arising at chronically photo-exposed sites. In other words, the data are compatible with a model in which older patients tend to present with thicker, more proliferative tumours as a consequence of senescence and possible diagnostic delay, rather than the de novo pathway itself conferring an independent, aggressive phenotype. Because this is an association within a retrospective design, causation cannot be established; the principal apparent drivers—age and cumulative solar damage—should be tested in prospective, adequately powered studies [6,7,8,26,27].

The molecular landscape of DNM and NAM further supports the CSD/non-CSD dichotomy observed in our clinical data. UV-induced mutational signatures—including C>T transitions at dipyrimidine sites and CC>TT tandem substitutions—are enriched in CSD-associated melanomas, which correspond predominantly to the DNM profile at chronically photo-exposed sites. *NF1* loss-of-function and *TERT* promoter mutations are molecular hallmarks of CSD-pathway melanomas, whereas *BRAF* V600E, arising via a UV-independent mechanism, is the signature mutation of non-CSD/NAM-associated melanomas and aligns with a younger age at diagnosis and truncal distribution observed in our NAM cohort [9,10]. *NRAS* mutations occur across both groups at intermediate frequencies. Molecular profiling was not available in the present retrospective series; its integration in future work would allow post hoc validation of the histological NAM/DNM classification, particularly in thick tumours where the nevus component may be obliterated, and would enable prognostic subgroup analysis beyond the clinicopathological variables available here.

A further consideration relevant to the interpretation of our classification is the “overgrowth” phenomenon: rapidly expanding invasive melanomas may completely obliterate the residual benign nevus component during vertical growth, leaving no histological trace of the precursor. In such cases, tumours representing true NAMs may be classified as DNM because no nevus remnant is detectable on standard sections. This phenomenon disproportionately affects thick tumours and likely contributes to underestimation of the true NAM prevalence in our cohort, as in retrospective histological series generally [28]. Awareness of this mechanism is important when interpreting the DNM:NAM ratio and the histopathological features attributed to the de novo group.

Solar elastosis—defined histologically as dermal accumulation of degraded elastic fibres reflecting chronic UV exposure—is a hallmark of lentigo maligna/LMM and serves as a tissue-level proxy for cumulative UV damage and the CSD pathway. Its presence in non-LMM subtypes, particularly SSM and NM arising at chronically photo-exposed anatomical sites, has been documented and is consistent with the CSD-driven melanomagenesis pathway that our analysis associates predominantly with DNM [20].

### Future Directions

The present findings are hypothesis-generating and warrant confirmation in prospective, multi-centre studies. Future work should incorporate (i) molecular profiling (*BRAF*, *NRAS*, *NF1*, and *TERT*) to validate the histological NAM/DNM classification and characterise mutational landscape differences; (ii) long-term survival and recurrence data to determine whether the de novo vs. nevus-associated origin carries independent prognostic value after controlling for Breslow thickness and stage; (iii) standardised assessment of cumulative sun exposure, Fitzpatrick phototype, total nevus count, and atypical nevus burden; (iv) prospective recording of surveillance modality and detection context to assess the impact of dermoscopic follow-up on stage at diagnosis in NAM; and (v) external validation in multi-institutional Romanian and European cohorts to assess the generalisability of these findings beyond a single tertiary referral centre.

## 5. Conclusions

This study indicates that, although de novo melanomas frequently present with more aggressive clinicopathological features (greater Breslow thickness and higher Clark level), these characteristics appear to be driven principally by advanced patient age and tumour location rather than by an intrinsic, independent de novo phenotype. These findings challenge the conventional view of de novo melanoma as inherently more aggressive and suggest that the observed differences may largely reflect patient demographics and diagnostic intervals. Given the retrospective design, these associations are hypothesis-generating and warrant confirmation in prospective, multicentre cohorts incorporating molecular and survival data.

## 6. Limitations

This study has several limitations. First, the single-centre design, while ensuring consistent diagnostic criteria throughout the study period, may introduce selection bias arising from the referral patterns of a tertiary academic centre, which may overrepresent diagnostically complex, atypical, or advanced cases relative to the general melanoma population. Findings therefore require external validation in multi-institutional cohorts. The absence of long-term survival data precludes assessment of whether the de novo vs. nevus-associated origin carries independent prognostic value. Second, the histopathological distinction between DNM and NAM depends on the identification of residual nevus cells, which may be obscured in thick tumours or eliminated by the “overgrowth” phenomenon (rapid tumour growth obliterating the precursor nevus), potentially underestimating the true NAM proportion [28]. Reliance primarily on H&E staining in cases where immunohistochemistry was not applied may similarly result in a small number of NAMs being classified as DNM. Third, in the absence of systematically integrated clinico-dermoscopic correlation at the time of histological assessment, cases of NAM in which the nevus component was subtle or focal may have been classified as DNM, contributing to underestimation of NAM prevalence. Fourth, inter-observer concordance data for histological diagnoses are not available for this retrospective cohort, representing an inherent limitation of retrospective studies using archival pathology data. Fifth, clinical phenotypic variables—including Fitzpatrick phototype, total and atypical nevus count, and objective photodamage assessment—were not available in this retrospective dataset, precluding a full risk-factor analysis. Some subgroups (notably acral melanoma, n = 16) were small, limiting the precision of subtype-specific estimates. Molecular profiling was not available and should be integrated in future work.

## Figures and Tables

**Table 1:** Demographic characteristics and anatomic location of de novo (DNM) and nevus-associated melanoma (NAM).

Variable	De Novo, n (%)	Nevus-Associated, n (%)	*p*-Value
Age, years—median	66 [53–74]	56 [44–66]	<0.001 a
Age, years—mean ± SD	63.9 ± 15.1	55.8 ± 15.2	
Sex—Male	245 (53.7)	63 (50.8)	0.634 b
Sex—Female	211 (46.3)	61 (49.2)	
Anatomic location:			0.0006 b
Head & neck	111 (24.3)	12 (9.7)	
Trunk	206 (45.2)	79 (63.7)	
Upper extremities	54 (11.8)	8 (6.5)	
Lower extremities	79 (17.3)	23 (18.5)	
Other	6 (1.3)	2 (1.6)	

a Mann–Whitney U test; b χ^2^ test; SD, standard deviation.

**Table 2:** Histopathological characteristics of de novo (DNM) and nevus-associated melanoma (NAM).

Variable	De Novo	Nevus-Associated	*p*-Value
Breslow thickness, mm—median	2.00 [0.70–5.00]	1.25 [0.69–3.12]	0.021 a
Clark level—median	3 [2.75–4.00]	3 [2.00–4.00]	0.040 a
Mitotic rate, /mm^2^—median	3 [1–9]	2 [1–6]	0.049 a
Ulceration—n (%)	195 (42.8)	43 (34.7)	0.128 b
Regression—n (%)	143 (31.4)	33 (26.6)	0.363 b
Perineural invasion—n (%)	9 (2.0)	3 (2.4)	0.726 c

a Mann–Whitney U test; b χ^2^ test; c Fisher’s exact test. Median values expressed as median [IQR].

**Table 3:** Solar elastosis by anatomical site and histological subtype.

Histological Subtype	Head & Neck	Upper Ext.	Trunk	Lower Ext.	Total
**SSM**	20/43 (47%)	2/21 (10%)	15/174 (9%)	4/42 (10%)	47/305 (15%)
**Nodular melanoma**	15/38 (39%)	3/11 (27%)	6/51 (12%)	1/15 (7%)	27/124 (22%)
**Lentigo maligna/LMM**	8/9 (89%)	—	2/2 (100%)	—	12/13 (92%)
**Acral lentiginous**	—	0/2 (0%)	0/2 (0%)	0/18 (0%)	0/23 (0%)
**Melanoma in situ**	2/6 (33%)	—	1/10 (10%)	0/1 (0%)	3/17 (18%)
**Other**	8/16 (50%)	0/5 (0%)	4/42 (10%)	0/9 (0%)	17/83 (20%)
**Total**	53/112 (47.3%)	5/39 (12.8%)	28/281 (10.0%)	5/85 (5.9%)	106/565 (18.8%)

SSM, superficial spreading melanoma; LMM, lentigo maligna melanoma. Denominators represent cases in which solar elastosis was explicitly documented in the pathology report. Cases with anatomical sites not specified in the report (n = 48) are included in the column totals. χ^2^ across the four main anatomical regions (head/neck, upper extremities, trunk, lower extremities) = 88.2; df = 3; *p* = 5.4 × 10^−19^; head and neck vs. trunk: OR = 8.1, *p* = 3.3 × 10^−15^ (Fisher’s exact test).

**Table 4:** Multivariable logistic regression model for predictors of de novo melanoma (reference: nevus-associated melanoma).

Predictor	β (log)	Odds Ratio (95% CI)	*p*-Value
Age	0.032	1.03 (1.02–1.05)	<0.001
Sex (Male vs. Female)	−0.114	0.89 (0.56–1.41)	0.621
Location: Head & neck	1.006	2.73 (1.39–5.39)	0.004
Location: Upper limbs	0.943	2.57 (1.13–5.81)	0.024
Location: Lower limbs	0.239	1.27 (0.72–2.24)	0.409
Location: Trunk	Reference	—	—
Histology: Nodular	0.505	1.66 (0.81–3.37)	0.164
Histology: Acral	1.341	3.82 (0.47–30.8)	0.209
Histology: Other	0.394	1.48 (0.29–7.47)	0.633
Histology: SSM	Reference	—	—
Breslow thickness	0.016	1.02 (0.95–1.09)	0.644
Mitotic rate	0.022	1.02 (0.99–1.06)	0.199

CI, confidence interval; SSM, superficial spreading melanoma. The wide confidence interval for acral histology reflects the small acral subgroup (n = 16).

## Data Availability

The data supporting the reported results are not publicly available due to patient privacy and ethical restrictions. Requests may be directed to the corresponding author.

## Abbreviations

The following abbreviations are used in this manuscript:

CSD	Chronic sun damage
DNM	De novo melanoma
GDPR	General Data Protection Regulation
IQR	Interquartile range
NAM	Nevus-associated melanoma
OR	Odds ratio
SSM	Superficial spreading melanoma
UV	Ultraviolet
